# Metabolite profiles of medulloblastoma for rapid and non-invasive detection of molecular disease groups

**DOI:** 10.1016/j.ebiom.2023.104958

**Published:** 2024-01-06

**Authors:** Sarah Kohe, Christopher Bennett, Florence Burté, Magretta Adiamah, Heather Rose, Lara Worthington, Fatma Scerif, Lesley MacPherson, Simrandip Gill, Debbie Hicks, Edward C. Schwalbe, Stephen Crosier, Lisa Storer, Ambarasu Lourdusamy, Dipyan Mitra, Paul S. Morgan, Robert A. Dineen, Shivaram Avula, Barry Pizer, Martin Wilson, Nigel Davies, Daniel Tennant, Simon Bailey, Daniel Williamson, Theodoros N. Arvanitis, Richard G. Grundy, Steven C. Clifford, Andrew C. Peet

**Affiliations:** a Institute of Cancer and Genomic Sciences, University of Birmingham, Birmingham, UK; b Birmingham Children’s Hospital, Birmingham, UK; c Wolfson Childhood Cancer Research Centre, Newcastle University Centre for Cancer, Translational and Clinical Research Institute, Newcastle University, Newcastle upon Tyne, UK; d Department of Applied Sciences, Northumbria University, Newcastle upon Tyne, UK; e Children’s Brain Tumour Research Centre, Queen’s Medical Centre, University of Nottingham, Nottingham, UK; f Radiological Sciences, Division of Clinical Neuroscience, University of Nottingham, Nottingham, UK; g Sir Peter Mansfield Imaging Centre, University of Nottingham, Nottingham, UK; h Alder Hey Children’s Hospital, Liverpool, UK; i University of Liverpool, Liverpool, UK; j RRPPS, University Hospital Birmingham, Birmingham, UK; k Institute of Metabolism and Systems Research, University of Birmingham, UK; l Department of Electronic, Electrical and Systems Engineering, University of Birmingham, UK

**Keywords:** Medulloblastoma, Groups, Metabolites, Metabolomics, Mass spectrometry, Radiology

## Abstract

**Background:**

The malignant childhood brain tumour, medulloblastoma, is classified clinically into molecular groups which guide therapy. DNA-methylation profiling is the current classification ‘gold-standard’, typically delivered 3–4 weeks post-surgery. Pre-surgery non-invasive diagnostics thus offer significant potential to improve early diagnosis and clinical management. Here, we determine tumour metabolite profiles of the four medulloblastoma groups, assess their diagnostic utility using tumour tissue and potential for non-invasive diagnosis using *in vivo* magnetic resonance spectroscopy (MRS).

**Methods:**

Metabolite profiles were acquired by high-resolution magic-angle spinning NMR spectroscopy (MAS) from 86 medulloblastomas (from 59 male and 27 female patients), previously classified by DNA-methylation array (WNT (*n = 9*), SHH (*n = 22*), Group3 (*n = 21*), Group4 (*n = 34*)); RNA-seq data was available for sixty. Unsupervised class-discovery was performed and a support vector machine (SVM) constructed to assess diagnostic performance. The SVM classifier was adapted to use only metabolites (*n = 10*) routinely quantified from *in vivo* MRS data, and re-tested. Glutamate was assessed as a predictor of overall survival.

**Findings:**

Group-specific metabolite profiles were identified; tumours clustered with good concordance to their reference molecular group (93%). GABA was only detected in WNT, taurine was low in SHH and lipids were high in Group3. The tissue-based metabolite SVM classifier had a cross-validated accuracy of 89% (100% for WNT) and, adapted to use metabolites routinely quantified *in vivo*, gave a combined classification accuracy of 90% for SHH, Group3 and Group4. Glutamate predicted survival after incorporating known risk-factors (HR = 3.39, 95% CI 1.4–8.1, p = 0.025).

**Interpretation:**

Tissue metabolite profiles characterise medulloblastoma molecular groups. Their combination with machine learning can aid rapid diagnosis from tissue and potentially *in vivo*. Specific metabolites provide important information; GABA identifying WNT and glutamate conferring poor prognosis.

**Funding:**

10.13039/501100001273Children with Cancer UK, 10.13039/501100000289Cancer Research UK, Children’s Cancer North and a 10.13039/501100000774Newcastle University PhD studentship.

**Research in context**

**Evidence before this study**

Medulloblastoma molecular groups have clinical and prognostic significance and are increasingly used in disease diagnostics and treatment stratification. Currently group diagnosis is obtained from tissue histological and molecular analyses and takes 3–4 weeks, risking tumour progression prior to the initiation of a definitive management plan. Metabolite profiles combined with machine learning provide a diagnostic tool for common childhood brain tumours both *in vivo* and *ex vivo*. A previous *in vivo* study has shown the potential for metabolite profiles to detect differences between medulloblastoma groups. Specific metabolites have been shown to have potential prognostic value in children’s brain tumours, with medulloblastoma glutamate levels identified as a marker of poor prognosis in a small single centre study.

**Added value of this study**

We have determined metabolite profiles from a group of 86 clinically and molecularly well characterised medulloblastoma tissue samples using high-resolution magic-angle spinning nuclear magnetic resonance spectroscopy (MAS). Significant differences in multiple metabolites exist between groups, with GABA being a strong predictor of the WNT group. A machine learning diagnostic classifier developed for the medulloblastoma groups gives a high level of diagnostic accuracy and could provide a rapid diagnostic tool. This classifier was adapted to assess its potential for use on routine *in vivo* MRS data giving a non-invasive diagnosis of group. Tumour glutamate levels were confirmed prospectively as a strong predictor of poor prognosis in medulloblastoma, with other metabolites identified as having a potential prognostic significance.

**Implications of all the available evidence**

Metabolite profiles are strong characteristics of tumour type in childhood brain tumours and can identify medulloblastoma molecular groups as well as providing prognostic information. Metabolite profiles can be determined rapidly from *ex vivo* tumour tissue and also non-invasively using an MRI scanner. This has important clinical implications, enabling early diagnosis, clinical planning and management. The principles established will be applicable to other tumour types and age groups.

## Introduction

Medulloblastoma is the most common malignant embryonal brain tumour of childhood. Disease-wide five-year survival rates have improved to around 70% with the introduction of treatments stratified by clinical, imaging and histological criteria; age, metastatic stage, histological subtype and extent of surgical resection represent the major risk-factors.^1^ Importantly, distinct molecular disease groups are recognised (WNT, SHH, Group3 and Group4), each with characteristic clinical, pathological and molecular features,^2^ which underpin the current WHO disease classification.^3^

Molecular disease groups increasingly form the basis of treatment stratification in contemporary clinical trials of risk-adapted and targeted therapies (e.g. therapy reduction for favourable-risk WNT tumours^4^; SMO inhibitors for SHH tumours^5^; SHH trials^6^). Molecular genetic methods applied to surgical material represent the current diagnostic ‘gold-standard’ for molecular group detection.^3^ However, these approaches have significant limitations, with their dependence on surgical material and typically long turnaround times combining to give 3–4 weeks from presentation to full diagnosis^7^; and consequent incompatibility with early planning of treatment and/or surgical strategy, or non-invasive disease management.

The development of robust non-invasive methods for timely medulloblastoma grouping offers the potential to support improved disease management, through adaptations such as rapid diagnosis and prognostication, early planning and refinement of treatment, as well as informing discussions with the family in the crucial early stages of their clinical management. Tumour metabolite profiles^8^ may be detected through routine diagnostic imaging, such as magnetic resonance spectroscopy (MRS).^9^^,^^10^ We and others have previously demonstrated metabolite profiles can distinguish medulloblastomas from other major childhood brain tumour types arising in the cerebellum with high accuracy, using *in vivo* MRS and classification by machine learning.^11^^,^^12^ Moreover, initial evidence has indicated medulloblastoma disease groups have distinct spectral features,^13^ and we have identified tumour metabolites with prognostic potential in medulloblastoma (e.g. high glutamate; poor prognosis).^14^ These studies provide first proof-of-principle of the clinical potential of metabolite profiling in medulloblastoma, however any wider utility for disease grouping remains to be established.

Developing a non-invasive test directly from *in vivo* MRS presents several challenges, particularly around collection of sufficient numbers of cases and associated imaging data representative of the different molecular groups, from across multiple centres. To circumvent these, we have developed methods for metabolite profiling in *ex vivo* tissue samples, termed high-resolution magic angle spinning NMR (MAS), which allow the selection of representative snap-frozen samples for analysis from tumour banks, followed by use of this information to develop *in vivo* MRS methods.^15^ Notably, tissue MAS measures a greater range of metabolites than detected by standard *in vivo* MRS – thus allowing greater potential for discovery and characterisation, while closely mirroring those metabolite concentrations detected in common between the two techniques.^16^^,^^17^ Importantly, we have demonstrated that *ex vivo* metabolite profiles can distinguish between closely related tumour groups^16^^,^^18^

Here, we applied these techniques to characterise the metabolite profiles of the medulloblastoma molecular disease groups. Using machine learning, we constructed discriminatory classifiers which can be developed into diagnostic tests, applicable in both *ex vivo* tissue and *in vivo* spectroscopy analysis, as a basis for rapid and/or non-invasive testing. Further, we prospectively validated glutamate as a biomarker of poor prognosis, for use alongside diagnostic classifiers. Together, these findings establish metabolite profiles as a strong characteristic of medulloblastoma groups forming the basis of rapid and non-invasive tests to support improved disease management.

## Methods

Molecular profiling was undertaken on 86 clinically annotated medulloblastomas, collected from UK Children’s Cancer and Leukaemia Group (CCLG) and collaborating centres (Supplementary Table S1). ‘Gold-standard’ molecular group status (WNT (*n = 8*), SHH (*n = 22*), Group3 (*n = 21*), Group4 (*n = 34*)) was determined by 450K DNA methylation profiling (Illumina) of formalin-fixed paraffin-embedded (FFPE) or frozen tissue samples, and our established validated classifiers, as previously described^19^; one further case did not have methylation profiling but was WNT on local immunohistochemistry and was included only in unsupervised analyses. In addition, validated immunohistochemical, mutational and copy number correlates of specific groups (e.g. *CTNNB1* mutation and chromosome 6 loss in WNT tumours) were assessed where possible,^19^ RNA-seq analysis and subsequent group assignment was undertaken on a subset of 60 tumours as previously described.^20^ No preliminary data was available to determine sample size, but studies of a similar nature have successfully used cohorts with about 20 cases per disease group to discriminate between classes, with fewer being reasonable in tumour types which are expected to have very different characteristics.^15^

*Ex vivo* tissue was snap frozen after surgery and stored at −80 °C. Metabolite profiles were generated for the 86 medulloblastoma cases using MAS and, as a comparator, post-mortem cerebellar tissue (*n = 7*), ependymoma (*n = 18*) and pilocytic astrocytoma (*n = 24*).^15^ TMSP was added to the samples as a ppm reference. Data was acquired using a 500 MHz Bruker Avance spectrometer (Bruker, Coventry, UK) fitted with a MAS probe. The rotor was spun at 4.8 KHz at a temperate of 278 K. A NOESY pulse sequence was used with 2s water pre-saturation. Fourier transformed data was imported into MestReNova 9.0.1 software suite (Mestrelab Research, Spain) for metabolite assignment and quantification, with 26 metabolite and 5 lipid macromolecule values being quantified (Supplementary Table S2). The concentrations for all the metabolites excluding lipids determined from the MestReNova analysis in arbitrary units were summed to provide a normalization factor. All metabolite and lipid values are then reported as the arbitrary value divided by this normalization factor. We refer to these values as normalized metabolite concentrations. Mean centering and scaling by standard deviation of these normalized metabolite concentrations was undertaken prior to use in machine learning. Absolute concentrations were not determined.

### Ethics

This study has Research Ethics Committee approval (Trent Multi-Centre Research Ethics Committee East Midlands-Derby, 04/MRE04/41, 25 August 2004) and CCLG Biological Studies Committee approval (2015BS05)). Informed consent was obtained from the patient or parent/legal guardian.

### Statistics

Class discovery was undertaken by unsupervised analysis of the MAS profiles using the R statistical package (R3.61, R Core Team, www.R-project.org), initially by principal component analysis, selection of the principal components accounting for 90% of the variance and then divisive hierarchical Cluster analysis. Univariate analysis of differences between groups was undertaken using Kruskal–Wallis tests with a Bonferroni correction and post-hoc Mann–Whitney U tests and post-hoc Dunn Tests. A ROC analysis was used to assess diagnostic potential of the individual metabolites with confidence intervals determined by 2000 bootstraps. A Gene Set Enrichment Analysis (GSEA) was undertaken to determine gene sets mapped to the KEGG dataset. Individual gene expression to metabolite correlations for specific metabolites were evaluated using Spearman’s correlation. Machine learning classifiers were built using a support vector machine (SVM) with metabolite concentrations as the input and 10-fold cross validation undertaken. Survival analysis was undertaken using univariate and multivariable Cox regression on normalised metabolite concentrations and known risk factors to determine the association of metabolites with survival. The Cox regression assumptions were tested, proportionality was tested by log–log survival curves and the Schoenfeld global test, linearity was tested using penalised spline regression. Log-rank tests were used to determine significance of Cox models. The start time was taken as the date of diagnosis and the end time as either date of death or date of last recorded follow-up if patient was alive, death was taken as the event and those alive were censored with the date being final follow-up prior to study end. The origin was taken as the start time. The variables for the multi-variate Cox regression were selected on prior knowledge to be those currently used in clinical practice as this is more relevant than selecting variables using a data based variable selection algorithm. Only one metabolite, glutamate, was selected, since this was reported to have prognostic value in a previously published study.

### Role of funders

The funders of the study had no role in study design, data collection, data analysis, data interpretation, or writing of the report. The corresponding authors had full access to all of the data and had the final responsibility to submit for publication.

## Results

### Unsupervised classification of medulloblastoma groups using MAS profiles

**Fig. 1:**
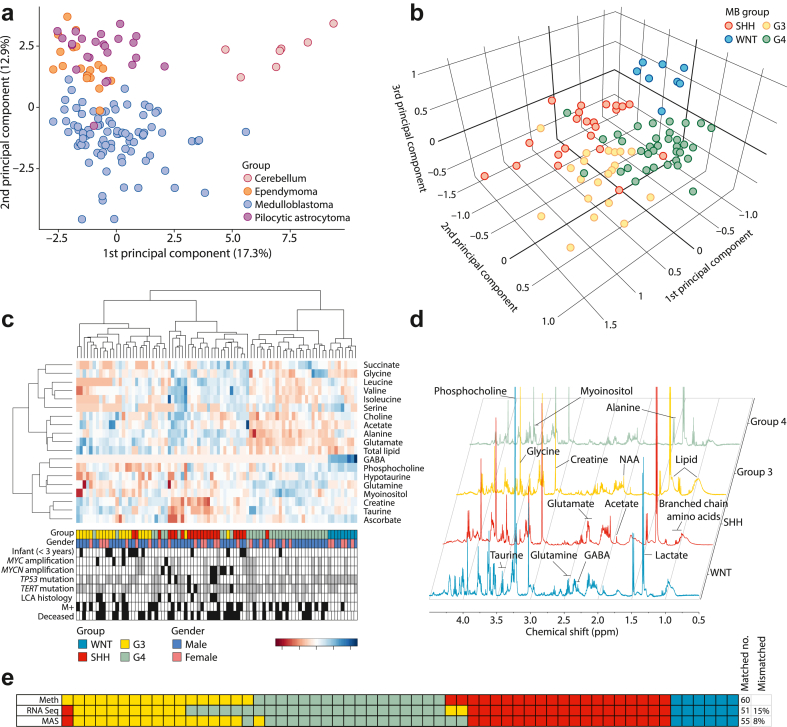
**Tissue metabolite profiles distinguish cerebellar tumour types and medulloblastoma groups. a**) PCA scatterplot shows tissue type-specific metabolite profiles capable of separating medulloblastoma tumours (n = 86) from other posterior fossa tumours (pilocytic astrocytoma (n = 24), ependymoma (n = 18)) and normal cerebellar tissue samples (n = 7). **b**) Metabolite profiles are capable of separating the four main molecular groups of medulloblastoma through unsupervised PCA analysis. **c**) Unsupervised hierarchical clustering further validates and illustrates clustering of metabolite profiles by group, sub-structures of the data, and variations in metabolite concentrations between groups. Black boxes show tumours positive for the feature of interest, grey boxes indicate desmoplastic/nodular pathology (LCA histology track) or data not available (all other tracks). **d**) Annotated representative spectra show visual differences between the 4 groups. **e**) Alignment of unsupervised MRS classification and RNA-seq-based classification with ‘gold standard’ DNA methylation array-based classification shows tissue metabolite profiles perform equivalently to RNA-seq. Colour code for molecular groups applies to all part of the figure. G3, Group3; G4, Group4; LCA, large-cell/anaplastic.

A principal component analysis (PCA) of MAS tissue metabolite profiles from post-mortem cerebellar tissue, medulloblastoma, ependymoma and pilocytic astrocytoma showed cerebellar tissue is clearly distinguished from the tumours and medulloblastomas form a cluster largely separate from the other tumour types (Fig. 1a). A further PCA performed on the tissue metabolite profiles from WNT, SHH, Group3 and Group4 medulloblastomas shows clustering by molecular group (Fig. 1b). Supporting the PCA data, unsupervised hierarchical clustering identifies four clusters which map closely to the four medulloblastoma molecular groups, and allows the identification of key metabolites that vary between them (Fig. 1c). In particular, GABA is high in WNT tumours and absent in the other three groups. Total lipid concentration is highest in the cluster corresponding to Group3. Glutamate is highest in the clusters associated with SHH and Group3. Some of these features can be appreciated visually in the MAS spectra; representative spectra labelled with the key metabolites are shown in Fig. 1d.

Of the 86 samples present in the MAS cohort, 60 were also profiled using RNA-seq. Using a reference classification for molecular groups derived from hundreds of DNA methylation arrayed samples,^19^ we compared the accuracy of unsupervised MAS classification to that from RNA-seq for this cohort (Fig. 1e). When the RNA-seq and metabolomic group calls were aligned and compared to the gold standard DNA methylation array group assignments, the outcome was remarkably similar in terms of accuracy between the two datasets (Fig. 1e). Despite the limitation that MAS quantifies only 30 metabolite and lipid macromolecule values compared to the 1000s of genes sampled by RNA-seq, the proportion of discordant cases is similar between the methods, with 9 out of 60 (15%) for RNA-seq compared with 5 out of 60 (8%) for metabolite profiles, and MAS improving the assignment between Group3 and Group4. The same samples switch between SHH and Group3/4 on RNA-seq and metabolomic classification.

### Metabolite levels differ between medulloblastoma disease groups and can discriminate between them

**Fig. 2:**
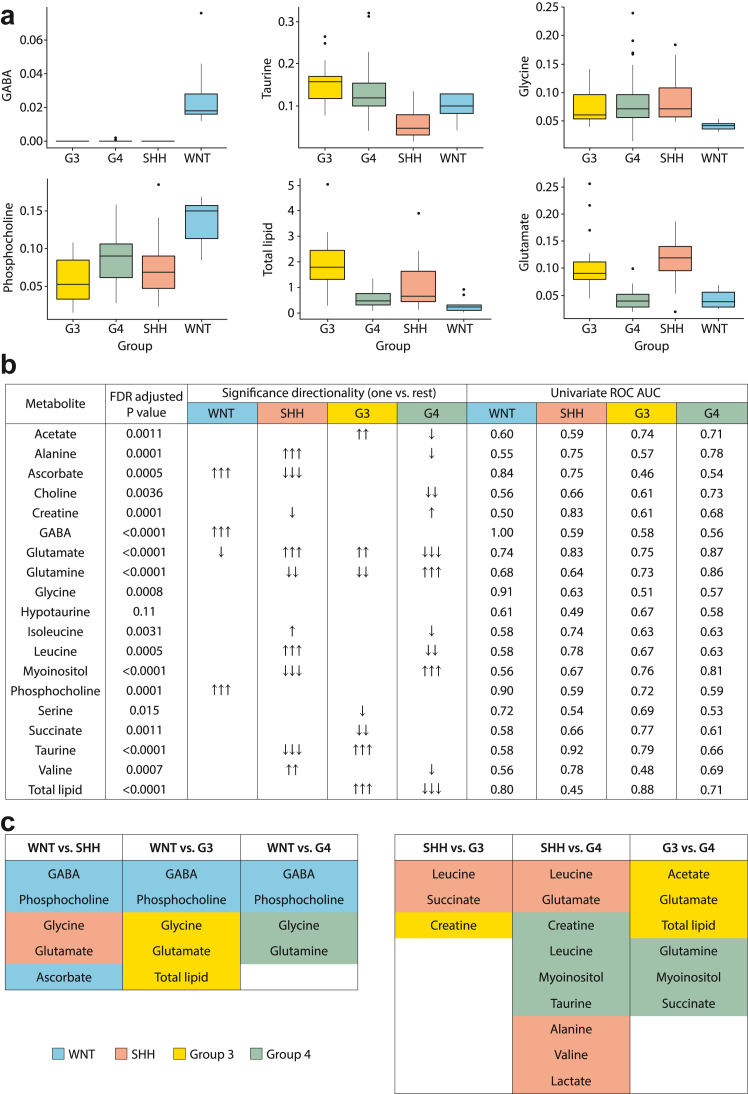
**Differences in metabolite profiles between molecular groups. a**) Box and whisker plots show differences in metabolite concentrations with a univariate ROC > 0.8; whiskers represent 1.5 times the inter-quartile range (IQR). **b**) Many metabolites show significant differences in concentration between the groups. Univariate ROC analysis identifies metabolites which act as discriminators for groups. False Discovery Rate adjusted p value < 0.05 one arrow; <0.005 two arrows; <0.0005 three arrows, **c**) Summary of differences between groups shown in a pairwise manner. Shaded metabolites are significantly higher in concentration in that group. G3, Group3; G4, Group4.

Differences in normalised metabolite concentrations and lipid quantities were observed between groups (Fig. 2, Supplementary Table S3). Notably, GABA, glutamate, total lipids and taurine have an FDR corrected p value < 0.0001. A univariate ROC analysis identified 11 individual metabolites with high (>0.8) group-specific discriminative potential (Fig. 2b, Supplementary Table S4). Importantly, GABA was found to be a perfect discriminator of WNT medulloblastoma with a ROC AUC of 1. In addition, the ROC analysis shows WNT tumours were also characterised by high normalised concentrations of phosphocholine and ascorbate and low normalised concentrations of glycine and lipids. SHH tumours were characterised by high levels of glutamate and low levels of taurine and creatine. Group3 tumours were characterised by high levels of lipids. Group4 tumours were characterised by high glutamine and myo-inositol and low glutamate. A summary of differences between groups in a pairwise manner is given in Fig. 2c.

### Correlating metabolite profiles with gene expression patterns

**Fig. 3:**
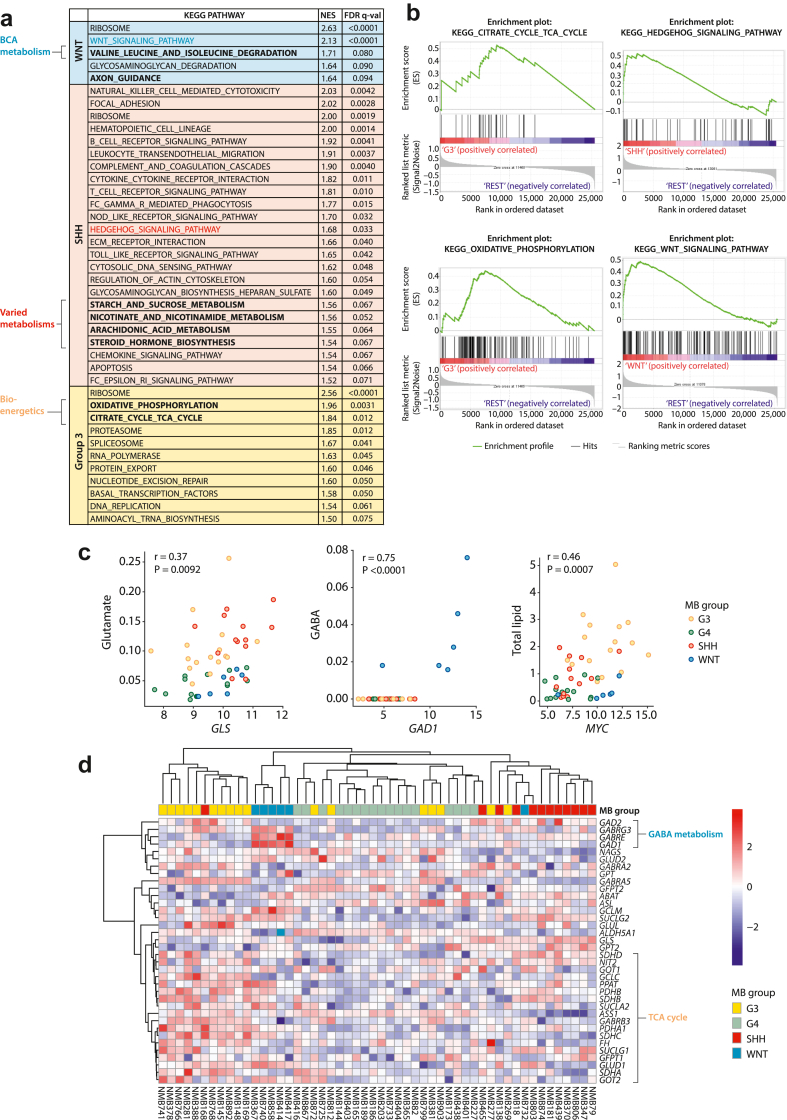
**Metabolic signatures in medulloblastoma RNA expression profiles. a,b**) GSEA analysis of pathways enriched in each medulloblastoma group using the KEGG pathway database (WNT: n = 6, SHH: n = 14, Group3: n = 17, Group4: n = 17). **a**) KEGG pathways significantly enriched in each group using gene set enrichment analysis (GSEA). There were no significantly enriched KEGG pathways in Group4. **b**). Enrichment plots for enriched metabolic pathways in each group. **c**) Differences in mRNA expression of metabolic enzymes are associated with alterations in concentration of their associated metabolites. **d**) Heatmap representing clustered mRNA expression levels of genes constituting the ‘Alanine, aspartate and glutamate metabolism’ KEGG pathway.

The relationship between gene expression and metabolite concentrations, and how this varies across groups, was next explored. A gene set enrichment analysis identified a number of KEGG pathways probed by MAS which were significantly enriched in the MB groups (Fig. 3a and b). These included valine, leucine, isoleucine degradation in WNT and the TCA cycle in Group3 and WNT tumours. Associations were therefore assessed between metabolite concentration and the expression of key biosynthetic enzymes and transporters (Fig. 3c). GABA concentration correlated significantly with expression of glutamate decarboxylase 1 (*GAD1*), an enzyme key to its synthesis, with both being particularly high in WNT tumours. The glutamine metabolising enzyme, glutaminase (*GLS*), and the main glutamine transporter, *SLC1A5*, were significantly positively correlated with each other whilst being significantly negatively correlated with glutamine (data not shown). In addition, glutamate was significantly positively correlated with *GLS*, which implies that glutamine to glutamate interconversion is an important regulator of the concentration of these metabolites in medulloblastoma. Group3 and SHH in particular show high glutamate and low glutamine associated with high *GLS* and *SLC1A5* expression. The expression of *BCAT1* (data not shown), the branched chain amino acid transaminase, correlated significantly with leucine and isoleucine concentration and was significantly higher in the SHH tumours. In addition, *MYC* expression was significantly correlated with total lipid across all groups. To further investigate gene expression in glutamate associated metabolic pathways, a heatmap representing clustered gene expression from genes included in ‘alanine, aspartate and glutamate metabolism’ from the KEGG (Kyoto Encyclopedia of Genes and Genomes) pathway database was undertaken and shows clustering by group (Fig. 3d).

### Constructing classifiers based on medulloblastoma tissue metabolite profiles

**Fig. 4:**
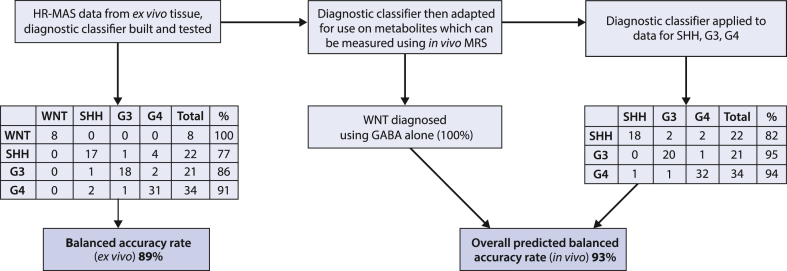
**Medulloblastoma disease group classification scheme and accuracy.** The HR-MAS classifier accuracy is obtained using 10-fold cross validation.

We next sought to develop diagnostic classifiers based on supervised machine learning of MAS profiles and assess their potential to classify using *in vivo* MRS data (Fig. 4). The strategy was first to develop a classifier based on all the MAS data available. The metabolites and lipid macromolecules quantified from the tissue samples were used to construct a support vector machine (SVM) classifier. A 10-fold cross validation was used to assess the performance of the tissue classifier, achieving a balanced accuracy rate of 89%. GABA is included in this model and all WNT tumours were classified correctly (Fig. 4).

### Assessing potential for non-invasive diagnosis using *in vivo* magnetic resonance spectroscopy

We then sought to adapt this classifier to make it appropriate for use with *in vivo* MRS data, and assess its potential to classify disease group using such datasets. Some metabolites quantified in tissue samples using MAS are not reliably quantifiable at clinical MRI scanner field strengths using conventional acquisition techniques. Therefore, a reduced input of 10 metabolite values corresponding to those commonly measured *in vivo* (alanine, creatine, glutamate, glutamine, glycine, myoinositol, N-acetyl aspartate, taurine, total choline, total lipid) was used to construct and test a second classifier for *ex vivo* MAS data in order to determine the potential impact of the reduced metabolite detection. The list of metabolites was determined from experience of *in vivo* MRS of children’s brain tumours including medulloblastoma with one exception, lactate was omitted since values in *ex vivo* tissue are prone to vary depending on tissue handing. Due to GABA acting as a marker for WNT tumours, neither GABA nor WNT tumours were included in this model. A 10-fold cross validation of this model achieved a balanced accuracy rate of 90% which, when combined with 100% accuracy for diagnosing WNT tumours, gives an overall combined balanced accuracy rate for the four groups of 93% (Fig. 4). For an *in vivo* MRS approach, the strategy would be for GABA to be used to identify WNT tumours and if not present then the classifier used to discriminate between the other three molecular groups.

### Prospective validation of tissue glutamate concentration as a prognostic marker in medulloblastoma

**Fig. 5:**
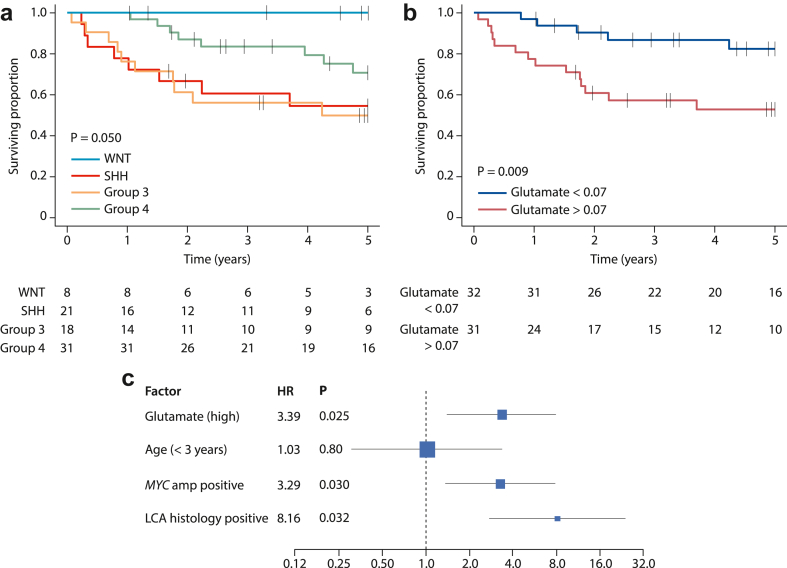
**Prognostic significance of glutamate in medulloblastoma. a**) Group-specific survival curves for all 78 patients with available data show WNT tumours have an excellent prognosis, whilst Group3 and SHH have poorer prognoses. **b**) Higher glutamate levels predict worse survival in 63 cases, following exclusion of those involved in the previous retrospective analysis which generated the hypothesis. p value derived using log-rank test. Life tables show the number at risk at each time point. **c**) Multi-variable analysis for whole group of 78 patients. LCA, large-cell/anaplastic histology.

As expected, analysis of 78 cases with survival information (Supplementary Table S1) showed molecular group to be a significant predictor of overall survival. The median follow-up time for all cases was 4.6 years (IQR 1.8–6.6, censoring: 63% survived to study end of which 63% were followed up for at least 5 years), WNT tumours had the best prognosis with no events up to 5 years. Group4 had an intermediate prognosis whilst Group3 and SHH had the worst prognosis (Fig. 5a). Excluding cases from a previous study^14^ gave a cohort of 63 (median follow-up time 4.2 years (IQR 0.7–6.7, censoring: 65% survived to study end of which 51% were followed up for at least 5 years) which showed that glutamate is a significant predictor of survival in cohort-wide analysis with a hazard ratio of 2.92 (95%CI 1.8–6.3, log rank 6.8, p = 0.009 Fig. 5b)). Testing of the Cox assumptions showed Global Schoenfeld Test p = 0.26, penalised splines regression non-linear term Chi squared 3.88, p = 0.27. Glutamate remained a significant predictor of survival after incorporating established clinical risk-factors (patient age at diagnosis, M stage, presence of large-cell/anaplastic pathology and *MYC/MYCN* amplification), with HR = 3.39 (95% CI 1.4–8.1, p = 0.025), thus independently validating and extending our previous observations (Fig. 5c). Exploratory survival analysis of the cohort of 78 cases identified further potential metabolite markers of prognosis (Supplementary Table S5).

## Discussion

We have shown metabolite profiles are a discriminating characteristic of the medulloblastoma disease groups. Combined with machine learning, these provide a powerful diagnostic tool exploitable for either rapid tissue diagnosis (MAS) or potentially as a non-invasive diagnostic aid (*in vivo* MRS). MAS can be acquired from small amounts of fresh tissue (10–50 mg) in <10 min, with potential use in intraoperative diagnosis. *In vivo* MRS is readily available and can provide a putative non-invasive diagnosis at the point of first clinical MRI scan.

Early diagnosis prior to analysis of tumour surgical material is highly pertinent to medulloblastoma. Early knowledge of tumour type and disease group gives opportunities for improved surgical planning and prognostication. This may be particularly useful in guiding surgical strategy for good prognosis tumours which are responsive to adjuvant therapies (e.g. WNT where there is invasion of the brain stem or infant SHH tumours).^6^ Early diagnosis further allows timely planning of adjuvant treatment, of particular importance to radiotherapy scheduling and, increasingly, logistical planning of proton therapies delivered away from the local centre. Critically, early informed discussions with patients and their families are possible, with benefits including reducing the initial period of clinical uncertainty and enabling the rapid instigation of genetic counselling in appropriate disease groups (i.e. around 40% of SHH patients have a germline involvement).^21^^,^^22^

DNA methylation-based classification derived from large independent cohorts represents the benchmark for group classifier accuracy,^6^ MAS and RNA-seq classification of our modestly-sized cohort showed good and equivalent concordance to this ‘gold-standard’ classification, with accuracy likely to improve further as the dataset increases and the MAS classifier is refined. The finding from tissue MAS data that GABA is WNT-specific provides a potential *in vivo* biomarker for this tumour type since GABA can be measured *in vivo* using specific MRS acquisition techniques (e.g. MEGA-PRESS).^23^^,^^24^ WNT could potentially be diagnosed by *in vivo* MRS with 100% accuracy. Reducing the number of metabolite quantities in the classifier to those readily determined clinically by *in vivo* MRS showed high accuracy of 90% in discrimination between the other 3 groups (SHH, Group3, Group4) indicating that non-invasive determination of medulloblastoma molecular groups by their metabolite profile is a realistic goal. A previous study of MRS in medulloblastoma showed more modest accuracy in determining molecular subtype than indicated by our data, but the study suffered from being based on a small dataset acquired historically and likely underestimates the potential accuracy of the technique.^13^ In terms of routine application, *in vivo* MRS is readily available in major centres with a field strength of 3 T becoming common and the acquisition techniques are commercially available. For GABA detection (WNT), MEGA-PRESS has recently become commercially available for use on clinical scanners.

Conventional MRI such as T1w and T2w, as well as more advanced MRI techniques such as diffusion weighted imaging, have been combined with machine learning to successfully discriminate between posterior fossa tumour types, notably medulloblastoma, pilocytic astrocytoma and ependymoma.^25^^,^^26^ Perfusion imaging has also been successful in grading of children’s brain tumours.^27^ Integration of metabolite-based disease grouping with conventional MRI and other advanced MR methods, offers opportunities to further secure and refine early diagnosis. Most notably, certain medulloblastoma disease groups are also associated with specific anatomical locations (e.g. SHH in the cerebellar hemispheres, WNT in the brainstem)^6^ and morphological appearances/enhancement characteristics (e.g. SHH tumours with DN pathology),^28^ which are readily discerned by conventional MRI. Radiomic features of conventional MRI have also been shown to be powerful characteristics of medulloblastoma molecular group.^29^ In addition to their diagnostic potential, metabolites show significant promise as prognostic biomarkers for medulloblastoma; we prospectively validated high glutamate, determined using *in vivo* MRS, as a marker of poor survival, and found GABA (WNT-associated), scylloinositol and valine to be associated with survival in a retrospective analysis of the metabolites. All now require further investigation in larger, clinically-controlled studies.

The acquisition of RNA-seq data together with MAS data allowed an initial exploration of putative mechanistic connections between the expression of enzymes related to metabolism and associated metabolite concentrations. The relatively small number of metabolites quantified here precluded an extensive integrated pathways analysis, and GSEA analysis of disease groups did not identify specific metabolic pathway correlates. Instead, we took the approach of investigating specific enzymes that have key relationships to metabolites found to be characteristic for specific groups, on a candidate basis. Notably, glutamate decarboxylase 1, which is involved in GABA synthesis, was strongly correlated with high GABA levels and a strong characteristic of WNT tumours, suggesting a mechanistic connection. Similarly, the significant correlation of glutamate to GLS levels, coupled with its association with a poor prognosis, indicates an important role for this enzyme The observation that glutamate is highest in clusters associated with SHH and Group3 corresponds with data in a study suggesting that TAp73 is a marker of glutamine addiction.^30^ Strong correlation between lipid levels and MYC expression confirm previous studies where MYC has been found to be involved in lipid metabolism.^31^ These initial findings underline the need to establish a more complete picture of medulloblastoma metabolism, encompassing more extensive metabolite profiles, larger cohorts and, as candidate pathways are identified, experimental investigations *in vitro* and *in vivo* disease models.

### Caveats and limitations

There are various aspects to the study design which should be understood when interpreting the results. In terms of the MAS technique and its processing, absolute concentrations are not calculated due to the lack of a reliable standard. When normalizing the concentrations, lipids were excluded for two reasons - whilst water soluble metabolites are in general easily attributable to a single molecule in MAS, lipid resonances are the result of many different molecules all with varying numbers of H atoms. From a practical point of view, lipids also have a large value which varies greatly between the samples. This combination of problems makes them a poor choice for use in normalization. The method of normalization used in this study is an approach taken in previous publications also allowing some consistency for comparison and reproducibility.^15^ There will be some quantitative differences between lipids measured by MAS and *in vivo*; this has not been addressed in the current study, but it would require further investigation when considering applying the classifier built on *ex vivo* MAS data to *in vivo* MRS data.

Although, absolute metabolite concentrations are not determined in this study, GABA concentrations can be estimated in relation to other metabolites, for example creatine which is readily determined in medulloblastoma spectra *in vivo*. The GABA concentrations in the WNT cases are approximately half those of the creatine concentrations. Allowing for the differences in proton number, the GABA resonances should be approximately a third the area of the creatine peak near 3 ppm, which should be readily identifiable.

In analyzing the MAS data, all peaks which were readily discernible were assigned to the appropriate metabolite and quantified. However, many other metabolites will be present at a concentration below that which can be detected by MAS. Additional metabolites could be measured using mass spectrometry which has greater sensitivity, but this technique is not so closely associated with *in vivo* MRS. MAS cannot be used to accurately quantify specific macromolecules of metabolic importance such a starch since they form broad peaks which merge into the baseline.

Whilst MAS offers the potential to provide an intraoperative diagnosis, there are various barriers to the application of this in routine clinical practice. MAS spectrometers are not readily available in hospitals at present, automated software for analysis including MAS data processing and machine learning would need to be developed, optimised and made available in an appropriate regulatory environment. However, prospective validation of the findings could provide the impetus to overcome these largely logistical barriers and are starting to be addressed in other clinical fields.^32^

This study does not present any *in vivo* MRS data and an application of the MAS findings to such data should be undertaken in the future. The main challenge of translating the findings of this study to *in vivo* MRS data is the lower accuracy to which the metabolites are determined by *in vivo* MRS than MAS of *ex vivo* samples. For example, challenges exist in discriminating glutamate and glutamine. However, there is already some evidence that this can be achieved in clinically acquired *in vivo* MRS^11^ and improved methods for MRS acquisition including higher field strength, better coil design and improved pulse sequences should further enhance accuracy. It is encouraging that an *in vivo* MRS study has already shown the potential to achieve discrimination between the medulloblastoma groups albeit at a lower accuracy than we report.^13^ Information from the *ex vivo* MAS data presented here should enable further improvement in the *in vivo* MRS classification accuracy. In this study, classifier accuracy was assessed using cross validation within the cohort and results should be further validated prospectively with these classifiers. Whilst *in vivo* MRS is available in most major centres, there is a need to develop and implement diagnostic classifiers which can be robustly tested across multiple centres taking into account variation in acquisition protocol and field strength.

The survival analysis suffers from the limited sample size which has precluded the testing of potential confounding variables in the multi-variable Cox model. In particular, the effect of subgroup should be tested with a larger cohort. The variables used in the multi-variable Cox analysis were selected to test the prior published observation of tumour glutamate being a marker of poor prognosis against the variables currently used in clinical practice to stratify treatment. This is a pragmatic approach, which we note could be prone to selection bias when determining hazard ratios. There is also a selection bias inherent to the hazard ratios since the hazard is calculated conditional on those who have survived. Metabolite concentrations differ greatly between the different groups and some metabolites are undetectable using MAS in some subgroups. This leads to an instability in the calculation of hazard ratios and their confidence intervals reported in Supplementary Table S5.

### Conclusion

Our work clearly demonstrates the power and potential of tissue metabolite profiles to aid in clinical management of medulloblastoma, through improved early diagnosis and prognostication, and intraoperative diagnostic testing. Moreover, our work provides important first insights into the importance of metabolism in the tumour biology and pathogenesis of medulloblastoma disease groups.

## Contributors

AP, RG and SC, designed and led the study. CB, FB, FS, HR, MA and SK did laboratory experimentation and analysis. AL, AP, CB, FB, DW, ECS, HR, LW, SK and TA did data handling and bioinformatics analysis. CB, FB and SK prepared figures. AP, DH, DT, DP, LMacP, LS, MW, ND, PSM, SA, SB, SC, SG and RG gathered samples, genomic, MRS and patient data, and provided interpretation. AP, CB, FB, SC and SK wrote the manuscript. All authors contributed to and approved the final manuscript. AP and SC have verified the underlying data.

## Data sharing statement

450 K DNA methylation array profiles used for the determination of human medulloblastoma molecular subgroup status are available at Gene Expression Omnibus accession number GSE93646. MAS data is available from the corresponding authors upon reasonable request.

## Declaration of interests

HR holds stock options in Healx (AI drug discovery in rare diseases). PSM is unpaid co-chair of SIOP-Europe Brain Tumour Group Imaging Group. We declare no other competing interests.
