# Supplementary material for: Metabolite profiles of medulloblastoma for rapid and non-invasive detection of molecular disease groups
Source: eBioMedicine. 2024 Jan 6;100:104958. doi: 10.1016/j.ebiom.2023.104958 (PMC10808898; doi:10.1016/j.ebiom.2023.104958)

**Supplementary Tables**

**Supplementary Table 1. Cohort information for the complete cohort.**

| **Tissue cohort** | **WNT** | **SHH** | **Group 3** | **Group 4** |
| --- | --- | --- | --- | --- |
| N | 9 | 22 | 21 | 34 |
| Sex (Male) | 4 | 14 | 14 | 27 |
| Age at diagnosis (Years)  Median  Range (IQR) | 8.6  7.1-10.1 | 6.3  3.1-12.7 | 4.8  2.5-8.3 | 6.1  5.0-10.2 |
| Histology  Classic  Desmoplastic Nodular  Extensive Nodularity  Large cell anaplastic  NOS | 7  0  0  0  1 | 6  3  2  5  6 | 12  0  0  5  4 | 24  0  0  0  10 |
| M+ | 1 | 5 | 10 | 17 |
| Resection status  GTR | 8 | 19 | 12 | 19 |
| *MYC/N* status  *MYC* amplified  *MYCN* amplified | 0  0 | 0  3 | 4  0 | 1  4 |
| *TP53* status  Mutation | 0 | 5 | 0 | 0 |

MAS cohort with survival information

| **Tissue cohort** | **WNT** | **SHH** | **Group 3** | **Group 4** |
| --- | --- | --- | --- | --- |
| N | 8 | 21 | 18 | 31 |
| Sex (Male) | 4 | 14 | 13 | 26 |
| Age at diagnosis (Years)  Median  Range (IQR) | 8.6  6.7-10.1 | 6.2  3.1-12.7 | 5.0  2.7-9.0 | 6.3  4.9-10.2 |
| Histology  Classic  Desmoplastic Nodular  Extensive Nodularity  Large cell anaplastic  NOS | 7  0  0  0  1 | 6  3  2  5  5 | 12  0  0  4  4 | 22  0  0  0  9 |
| M+ | 1 | 5 | 10 | 16 |
| Resection status  GTR | 8 | 18 | 12 | 18 |
| *MYC/N* status  *MYC* amplified  *MYCN* amplified | 0  0 | 0  3 | 4  0 | 1  4 |
| *TP53* status  Mutation | 0 | 4 | 0 | 0 |

**Supplementary Table 2. The ppm location and peak pattern for assigned metabolites.** Lactate was not included in the analysis due to lactate accumulation in the tissue over a variable amount of time during surgical excision.

| **Metabolite** | **^1^H chemical shift (ppm)** | **Multiplicity** |
| --- | --- | --- |
| Lipid | 0.90 | Broad singlet |
| Leucine (Leu) | 0.95 | Triplet |
| Isoleucine (Iso) | 1.01 | Doublet |
| Valine (Val) | 1.04 | Doublet |
| Lipid | 1.30 | Broad singlet |
| Lactate (Lac) | 1.33 | Doublet |
| Alanine (Ala) | 1.47 | Doublet |
| Lipid | 1.60 | Broad singlet |
| Acetate (Ace) | 1.92 | Singlet |
| N-acetylaspartate (NAA) | 2.02 | Singlet |
| N-acetylaspartylglutamate (NAAG) | 2.03 | Singlet |
| γ-amino butyric acid (GABA) | 2.30 | Triplet |
| Glutamate (Glu) | 2.35 | Multiplet |
| Succinate (Suc) | 2.41 | Singlet |
| Glutamine (Gln) | 2.45 | Multiplet |
| Glutathione (GSH) | 2.55 | Multiplet |
| Hypotaurine (hTau) | 2.65 | Triplet |
| Lipid | 2.80 | Broad singlet |
| Aspartate (Asp) | 2.82 | Doublet of doublets |
| Creatine (Cr) | 3.03 | Singlet |
| Choline (Cho) | 3.20 | Singlet |
| Phosphocholine (PCh) | 3.22 | Singlet |
| Glycerophosphocholine (GPC) | 3.23 | Singlet |
| Scyllo-inositol (sIns) | 3.34 | Singlet |
| Taurine (Tau) | 3.42 | Triplet |
| Myo-inositol (mIns) | 3.53 | Doublet of doublets |
| Glycine (Gly) | 3.56 | Singlet |
| Serine (Ser) | 3.84 | Doublet of doublets |
| Ascorbate (Asc) | 4.52 | Doublet |
| β D-glucose (Glc) | 4.65 | Doublet |
| Lipid | 5.30 | Broad singlet |

**Supplementary Table 3. Mean normalised metabolite concentrations for each subgroup.**

| Metabolite | Subgroup mean concentrations | | | | Kruskal Wallis  P value | FDR adjusted  P value | Post-hoc Dunn tests | | | | | |
| --- | --- | --- | --- | --- | --- | --- | --- | --- | --- | --- | --- | --- |
|  | WNT | SHH | Gr3 | Gr4 |  |  | WNT-SHH | WNT-Gr3 | WNT-Gr4 | SHH-Gr3 | SHH-Gr4 | Gr3-Gr4 |
| Acetate | 0.005 | 0.006 | 0.011 | 0.005 | 0.0008 | 0.0011 | 0.088 | 0.010 | 0.35 | 0.095 | 0.0070 | <0.0001 |
| Alanine | 0.044 | 0.055 | 0.044 | 0.029 | <0.0001 | 0.0001 | 0.11 | 0.491 | 0.022 | 0.066 | <0.0001 | 0.0033 |
| Ascorbate | 0.050 | 0.019 | 0.031 | 0.031 | 0.0002 | 0.0005 | <0.0001 | 0.010 | 0.0057 | 0.0079 | 0.0039 | 0.47 |
| Choline | 0.016 | 0.021 | 0.017 | 0.012 | 0.0032 | 0.0036 | 0.28 | 0.40 | 0.037 | 0.34 | 0.0005 | 0.0032 |
| Creatine | 0.065 | 0.043 | 0.078 | 0.075 | <0.0001 | 0.0001 | 0.018 | 0.23 | 0.15 | 0.0001 | <0.0001 | 0.38 |
| GABA | 0.027 | 0.000 | 0.000 | 0.000 | <0.0001 | <0.0001 | <0.0001 | <0.0001 | <0.0001 | 0.50 | 0.28 | 0.29 |
| Glutamate | 0.042 | 0.115 | 0.108 | 0.042 | <0.0001 | <0.0001 | <0.0001 | 0.0002 | 0.48 | 0.29 | <0.0001 | <0.0001 |
| Glutamine | 0.047 | 0.049 | 0.042 | 0.083 | <0.0001 | <0.0001 | 0.30 | 0.46 | 0.0002 | 0.21 | <0.0001 | <0.0001 |
| Glycine | 0.041 | 0.088 | 0.074 | 0.085 | 0.0005 | 0.0007 | <0.0001 | 0.0010 | <0.0001 | 0.121 | 0.24 | 0.27 |
| Hypotaurine | 0.019 | 0.017 | 0.011 | 0.018 | 0.11 | 0.11 | 0.20 | 0.023 | 0.31 | 0.065 | 0.30 | 0.15 |
| Isoleucine | 0.003 | 0.004 | 0.002 | 0.002 | 0.0026 | 0.0031 | 0.18 | 0.063 | 0.082 | 0.0007 | 0.0006 | 0.37 |
| Leucine | 0.008 | 0.012 | 0.005 | 0.006 | 0.0003 | 0.0005 | 0.12 | 0.037 | 0.077 | <0.0001 | 0.0001 | 0.26-1 |
| Myoinositol | 0.080 | 0.061 | 0.052 | 0.109 | <0.0001 | <0.0001 | 0.056 | 0.014 | <0.0001 | 0.20 | <0.0001 | <0.0001 |
| Phosphocholine | 0.137 | 0.073 | 0.057 | 0.088 | <0.0001 | 0.0001 | 0.0001 | <0.0001 | 0.0028 | 0.13 | 0.060 | 0.0034 |
| Serine | 0.015 | 0.007 | 0.004 | 0.006 | 0.014 | 0.015 | 0.061 | 0.0009 | 0.043 | 0.019 | 0.45 | 0.017 |
| Succinate | 0.002 | 0.003 | 0.001 | 0.002 | 0.0008 | 0.0011 | 0.044 | 0.12 | 0.010 | 0.0001 | 0.25 | 0.0004 |
| Taurine | 0.097 | 0.055 | 0.154 | 0.133 | <0.0001 | <0.0001 | 0.019 | 0.0059 | 0.66 | <0.0001 | <0.0001 | 0.064 |
| Valine | 0.006 | 0.010 | 0.007 | 0.006 | 0.0005 | 0.0008 | 0.010 | 0.35 | 0.29 | 0.0066 | <0.0001 | 0.10 |
| Total lipid | 0.308 | 1.056 | 1.953 | 0.522 | <0.0001 | <0.0001 | 0.041 | <0.0001 | 0.095 | .017 | 0.021 | <0.0001 |

**Table 4. AUC and confidence intervals (95%) for each metabolite.** Each group is tested against the rest combined.

| WNT |  |  |  |  |  |  |  |  |  |  |
| --- | --- | --- | --- | --- | --- | --- | --- | --- | --- | --- |
| Metabolite | Acetate | Alanine | Ascorabate | Choline | Creatine | GABA | Glutamate | Glutamine | Glycine | Hypotaurine |
| 5th centile | 0.40 | 0.38 | 0.72 | 0.41 | 0.34 | 1.00 | 0.62 | 0.51 | 0.85 | 0.46 |
| Median | 0.60 | 0.55 | 0.84 | 0.56 | 0.50 | 1.00 | 0.74 | 0.68 | 0.91 | 0.61 |
| 95th centile | 0.79 | 0.73 | 0.96 | 0.70 | 0.66 | 1.00 | 0.87 | 0.85 | 0.98 | 0.76 |
| Metabolite | Isoleucine | Leucine | Myoinositol | Phosphocholine | Scylloinositol | Serine | Taurine | Valine | Total Lipid |  |
| 5th centile | 0.38 | 0.43 | 0.40 | 0.80 | 0.44 | 0.52 | 0.43 | 0.38 | 0.65 |  |
| median | 0.58 | 0.58 | 0.56 | 0.90 | 0.61 | 0.72 | 0.58 | 0.56 | 0.80 |  |
| 95th centile | 0.78 | 0.74 | 0.72 | 0.99 | 0.78 | 0.92 | 0.73 | 0.75 | 0.95 |  |
|  |  |  |  |  |  |  |  |  |  |  |
| SHH |  |  |  |  |  |  |  |  |  |  |
| Metabolite | Acetate | Alanine | Ascorabate | Choline | Creatine | GABA | Glutamate | Glutamine | Glycine | Hypotaurine |
| 5th centile | 0.46 | 0.64 | 0.63 | 0.53 | 0.73 | 0.54 | 0.73 | 0.52 | 0.50 | 0.34 |
| Median | 0.59 | 0.75 | 0.75 | 0.66 | 0.83 | 0.59 | 0.83 | 0.64 | 0.63 | 0.49 |
| 95th centile | 0.72 | 0.86 | 0.87 | 0.79 | 0.93 | 0.63 | 0.94 | 0.76 | 0.76 | 0.64 |
| Metabolite | Isoleucine | Leucine | Myoinositol | Phosphocholine | Scylloinositol | Serine | Taurine | Valine | Total Lipid |  |
| 5th centile | 0.63 | 0.68 | 0.55 | 0.47 | 0.38 | 0.40 | 0.86 | 0.68 | 0.32 |  |
| Median | 0.74 | 0.78 | 0.67 | 0.59 | 0.51 | 0.54 | 0.92 | 0.78 | 0.45 |  |
| 95th centile | 0.85 | 0.88 | 0.79 | 0.72 | 0.65 | 0.67 | 0.98 | 0.88 | 0.59 |  |
|  |  |  |  |  |  |  |  |  |  |  |
| Group3 |  |  |  |  |  |  |  |  |  |  |
| Metabolite | Acetate | Alanine | Ascorabate | Choline | Creatine | GABA | Glutamate | Glutamine | Glycine | Hypotaurine |
| 5th centile | 0.60 | 0.44 | 0.32 | 0.47 | 0.46 | 0.54 | 0.65 | 0.61 | 0.38 | 0.51 |
| Median | 0.74 | 0.57 | 0.46 | 0.61 | 0.61 | 0.58 | 0.75 | 0.73 | 0.51 | 0.67 |
| 95th centile | 0.87 | 0.70 | 0.60 | 0.75 | 0.76 | 0.63 | 0.86 | 0.85 | 0.65 | 0.82 |
| Metabolite | Isoleucine | Leucine | Myoinositol | Phosphocholine | Scylloinositol | Serine | Taurine | Valine | Total Lipid |  |
| 5th centile | 0.49 | 0.51 | 0.61 | 0.59 | 0.42 | 0.57 | 0.69 | 0.34 | 0.80 |  |
| Median | 0.63 | 0.67 | 0.76 | 0.72 | 0.58 | 0.69 | 0.79 | 0.48 | 0.88 |  |
| 95th centile | 0.78 | 0.84 | 0.91 | 0.85 | 0.73 | 0.81 | 0.89 | 0.63 | 0.97 |  |
|  |  |  |  |  |  |  |  |  |  |  |
| Group4 |  |  |  |  |  |  |  |  |  |  |
| Metabolite | Acetate | Alanine | Ascorabate | Choline | Creatine | GABA | Glutamate | Glutamine | Glycine | Hypotaurine |
| 5th centile | 0.60 | 0.67 | 0.42 | 0.62 | 0.57 | 0.50 | 0.79 | 0.78 | 0.44 | 0.46 |
| Median | 0.71 | 0.78 | 0.54 | 0.73 | 0.68 | 0.56 | 0.87 | 0.86 | 0.57 | 0.58 |
| 95th centile | 0.82 | 0.89 | 0.67 | 0.85 | 0.80 | 0.63 | 0.95 | 0.94 | 0.70 | 0.70 |
| Metabolite | Isoleucine | Leucine | Myoinositol | Phosphocholine | Scylloinositol | Serine | Taurine | Valine | Total Lipid |  |
| 5th centile | 0.51 | 0.51 | 0.71 | 0.47 | 0.40 | 0.41 | 0.55 | 0.58 | 0.60 |  |
| Median | 0.63 | 0.63 | 0.81 | 0.59 | 0.52 | 0.53 | 0.66 | 0.69 | 0.71 |  |
| 95th centile | 0.74 | 0.75 | 0.90 | 0.71 | 0.65 | 0.65 | 0.78 | 0.81 | 0.82 |  |

**Supplementary Table 5. Survival analysis for cohort of 78 cases.**


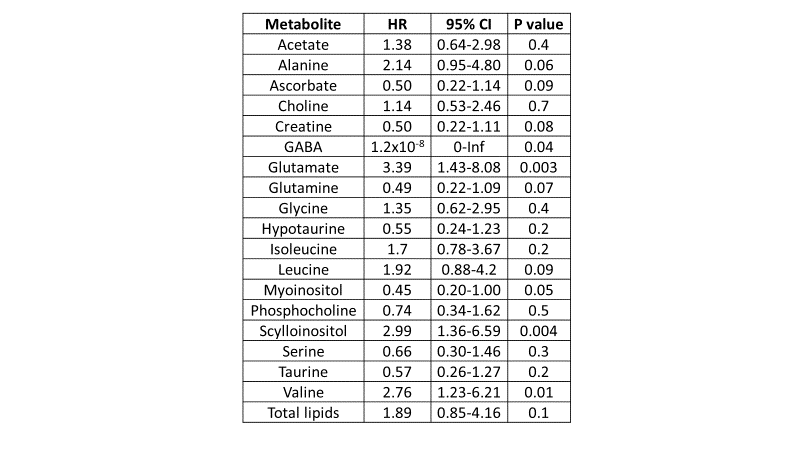

Supplement: Supplementary Tables S1–S5 [file mmc1.docx]
